# Chemotherapy‐induced peripheral neuropathy increases nontraumatic fracture risk in breast cancer survivors

**DOI:** 10.1002/jbm4.10519

**Published:** 2021-06-10

**Authors:** Brendan L. McNeish, James K. Richardson, Sarah G. Bell, Daniel G. Whitney

**Affiliations:** ^1^ Department of Physical Medicine and Rehabilitation Michigan Medicine Ann Arbor Michigan USA; ^2^ Department of Obstetrics and Gynecology University of Michigan Ann Arbor Michigan USA; ^3^ Institute for Healthcare Policy and Innovation University of Michigan Ann Arbor Michigan USA

**Keywords:** TUMOR INDUCED BONE DISEASE, OSTEOPOROSIS, FRACTURE PREVENTION, FRACTURE RISK ASSESSMENT, CANCER

## Abstract

Chemotherapy is a common treatment for breast cancer (BrCa) and can cause chemotherapy‐induced peripheral neuropathy (CIPN). CIPN contributes to falls, and is thus a potential risk factor for nontraumatic fractures (NTFx); yet, the effect of CIPN on NTFx risk has not been examined for BrCa survivors. We therefore investigated the association between CIPN and NTFx in BrCa survivors. Data were extracted from Optum's Deidentified Clinformatics® Data Mart Database years 2010–2015 in this retrospective cohort study. Among women, three groups were derived based on BrCa and CIPN status: BrCa+/CIPN+ (primary group of interest), BrCa+/CIPN− (first comparison group), and BrCa−/CIPN− (second comparison group). After propensity score matching the comparison groups to BrCa+/CIPN+ at a ratio of 1:11 (BrCa:control) for demographics, osteoporosis, glucocorticoid medication, comorbidities, and cancer‐related variables for BrCa+/CIPN−, 1‐year incidence rate (IR) of NTFx was determined for each group. The incident rate ratio (IRR) determined if the IR for NTFx was different for BrCa+/CIPN+ compared to BrCa+/CIPN− and BrCa−/CIPN−. Cox proportional hazards regression models estimated the hazard ratios (HRs) after adjusting for covariates that were unable to be matched for. The crude IR (95% confidence interval [CI]) of NTFx was 4.54 (2.32–6.77) for BrCa+/CIPN+ (*n* = 359), 2.53 (2.03–3.04) for BrCa+/CIPN− (*n* = 3949), and 1.76 (1.35–2.18) for BrCa−/CIPN− (*n* = 3949). The crude IRR of NTFx was significantly elevated for BrCa+/CIPN+ as compared to BrCa+/CIPN− (IRR = 1.80; 95% CI, 1.06–3.05) and BrCa−/CIPN− (IRR = 2.58; 95% CI, 1.50–4.44). The elevated rate of NTFx for BrCa+/CIPN+ remained unchanged after adjusting for aromatase inhibitors compared to BrCa+/CIPN− (HR = 1.79; 95% CI, 1.06–3.04). Female BrCa survivors have an increased 1‐year IR of NTFx after the onset of CIPN, suggesting that CIPN is an additive burden on NTFx risk among BrCa survivors. © 2021 The Authors. *JBMR Plus* published by Wiley Periodicals LLC on behalf of American Society for Bone and Mineral Research.

## Introduction

As of 2019, breast cancer (BrCa) survivors total 3.9 million and life expectancy continues to increase due to improvements in early detection and effective cancer treatments.^(^ ^1^ ^)^ BrCa treatments such as chemotherapy, radiation, and particularly endocrine therapy have been associated with bone loss, increasing the risk for nontraumatic fracture (NTFx), an indicator for bone fragility.^(^ ^2^, ^3^ ^)^ Importantly, NTFx may lead to higher risk of hospitalization and death in this population.^(^ ^4^ ^)^ The burden of NTFx among BrCa survivors underscores the importance of identifying and treating those most at risk for NTFx and the associated morbidity and mortality.

A common course of treatment for BrCa survivors is chemotherapy, often including neurotoxic agents such as taxanes, which are known to disrupt the function of the peripheral nervous system leading to chemotherapy‐induced peripheral neuropathy (CIPN).^(^ ^5^ ^)^ Peripheral neuropathy is a long‐standing risk factor for falls, fractures, and disability in the general population.^(^ ^6^, ^7^, ^8^ ^)^ Accordingly, CIPN in BrCa survivors is strongly associated with decreased mobility, increased falls, and increased disability.^(^ ^9^, ^10^ ^)^ Further, BrCa survivors with CIPN may reduce their physical activity due to their enhanced fall risk (e.g., fear of falling) and altered gait patterns,^(^ ^11^ ^)^ which would reduce weight‐bearing exercise and further contribute to bone fragility beyond fall risk alone. However, whether CIPN is a risk factor for NTFx among BrCa survivors has not been investigated. Addressing this knowledge gap could assist clinicians in identifying high risk subsets of the BrCa population for adjunct osteoporosis therapies and rehabilitation, thus improving bone and overall health.

A strong focus of survivorship care has been the identification of BrCa survivors at risk for cancer treatment–induced bone loss and subsequent NTFx. Currently, the guidelines from the American Society Cancer Organization (ASCO) and European Society for Medical Oncology (ESMO) both recommend screening of BrCa survivors for cancer treatment–induced bone loss.^(^ ^12^, ^13^ ^)^ It is recommended that risk factors for osteoporosis be reviewed, including aromatase inhibitor use, age, body mass index (BMI), physical activity, smoking, alcohol use, and a personal or family history of hip fracture.^(^ ^12^, ^14^ ^)^ However, CIPN is not among the ASCO or ESMO identified risk factors for NTFx, which is likely due to a lack of empirical evidence.^(^ ^12^ ^)^ Therefore, there is a need to determine if CIPN is a risk factor for NTFx among breast cancer survivors.

Nationwide administrative claims data provide an opportunity to examine the association between CIPN and acute NTFx risk among a large, heterogeneous population. Therefore, the purpose of this propensity score‐matched, observational cohort study was to leverage claims data to determine whether CIPN is associated with an increased 1‐year incidence rate of NTFx among women with BrCa. We hypothesized that women with newly diagnosed BrCa and CIPN (BrCa+/CIPN+) would have an increased 1‐year incidence rate of NTFx compared to women with newly diagnosed BrCa without CIPN (BrCa+/CIPN−) and women without BrCa and without CIPN (BrCa−/CIPN−).

## Materials and methods

### Data source

This study used claims data from the Optum's Deidentified Clinformatics® Data Mart Database (OptumInsight, Eden Prairie, MN, USA). This administrative claims data source is a national single private payer data base and contains medical and outpatient pharmacy data from beneficiaries that are covered by commercial or Medicare Advantage health plans in the United States, as described.^(^ ^15^ ^)^ In order to be enrolled, the beneficiary either pays for insurance coverage out of pocket, is covered by their employer, or is covered by their spouse that has employer‐based coverage that extends to family members. As a result, this sample may reflect a slightly more affluent sector of the general population and the findings from this study should be interpreted within the scope of this privately insured sample. Medical, procedure, and outpatient pharmacy claims from January 1, 2010 to December 31, 2014 (5 full calendar years) were used in this analysis. Because data are deidentified, the University Institutional Review Board approved this study as nonregulated.

All conditions, procedures, and medications were identified using codes and generic names of medications: International Classification of Diseases, Ninth Revision, Clinical Modification (ICD‐9‐CM) codes identified all medical conditions; Current Procedural Terminology (CPT) codes identified all procedures; and chemotherapy and BrCa treatment agents were identified using the generic names of relevant medications and relevant Healthcare Common Procedure Coding System (HCPCS) codes for non‐oral administered agents and are included in Supplementary Table S1.

### Sample selection

The primary group of interest was women with newly diagnosed BrCa that developed CIPN. Both BrCa and CIPN were identified by one or more claims that contained the ICD‐9‐CM codes for malignant (174.x) and nonmalignant (233.0) BrCa and CIPN (357.6), respectively. To identify incidence of CIPN in women with BrCa, the first claim of CIPN was identified in a 3‐year period from July 1, 2011 to June 30, 2014 among women with BrCa. Individuals were excluded if they had a claim for CIPN prior to the first CIPN claim from July 1, 2011 to June 30, 2014 in order to isolate incident cases. Because the majority of CIPN cases have been diagnosed approximately 6 months from the onset of initiating chemotherapy,^(^ ^16^ ^)^ the index date (i.e., start date of follow‐up) for the BrCa+/CIPN+ group was 6 months before their first CIPN claim date to better capture how chemotherapy may lead to acute risk of NTFx (i.e., 1 year).

In order to determine if the association of CIPN with one‐year incidence rate of NTFx is beyond the effect of BrCa, two groups were used for comparison: women with BrCa but without CIPN (BrCa+/CIPN−) and women without BrCa and without CIPN (BrCa−/CIPN−). For the BrCa+/CIPN− group, only incident BrCa cases were included to limit confounding by the effect of a newly diagnosed condition on risk of NTFx (e.g., change in lifestyle). Further, the index date for the BrCa+/CIPN+ and BrCa+/CIPN− groups were aligned with one another based on the time course of BrCa diagnosis and treatment. Specifically, the first BrCa claim, without a preceding BrCa claim, was identified from January 1, 2011 to September 31, 2013. As chemotherapy often starts approximately 3 months after the initial BrCa diagnosis,^(^ ^17^ ^)^ the index date for the BrCa+/CIPN− group was 3 months after the first BrCa claim date. For the second comparison group, BrCa−/CIPN−, women that had no claims for BrCa or CIPN and did not have exposure to chemotherapy were included, and their index date was randomly assigned in the calendar years 2011 to 2013 using a uniform distribution (visually inspected by author Daniel G. Whitney), as described.^(^ ^18^ ^)^

Following group allocation, women were included if they were: (i) ≥18 years of age; (ii) had ≥12 months of continuous health plan enrollment in the preindex period to ascertain baseline data, which is common practice for claims‐based research^(^ ^19^ ^)^; and (iii) had ≥1 day of follow‐up in the postindex period for incidence of NTFx to retain as much of the starting sample as possible given the ability of this study to account for censoring. Individuals were excluded if they had one or more fractures at any location and of any type (e.g., NTFx, trauma‐related fracture) in their preindex period, because sustaining a fracture increases risk for subsequent fracture regardless of energy level.^(^ ^20^ ^)^

### NTFx

Incidence of NTFx in the 1‐year postindex period was identified as a fracture that occurred at an unspecified site, vertebral column, hip (including proximal femur), non‐proximal femur, tibia/fibula, humerus, or ulna/radius that did not have a trauma code (e.g., fell from roof, car accident) 7 days before and after the fracture claim date, as described.^(^ ^21^, ^22^, ^23^, ^24^ ^)^ For those that did not sustain an NTFx in the 1‐year postindex period, individuals were right censored according to the day they discontinued health plan enrollment or end of study period (i.e., 1 year postindex), based on whichever occurred first.

### Covariates

Covariates were selected based on their relevance to CIPN, BrCa, NTFx, and availability and reliability in administrative claims databases. Age, race, and US region of residence from the time of index date, and whether the BrCa was malignant or nonmalignant was included. Chemotherapy exposure was determined as one or more outpatient pharmacy claims for any relevant chemotherapy agents or one or more medical claims for any relevant HCPCS codes for non‐oral administered chemotherapy. Chemotherapy included neurotoxic and non‐neurotoxic agents^(^ ^25^ ^)^ and are included Supplementary Table S1, and examined in this study as two non‐mutually exclusive variables: any chemotherapy, including both neurotoxic and non‐neurotoxic agents and neurotoxic agents. As guided by previous studies,^(^ ^26^, ^27^ ^)^ osteoporosis medications prescribed in the preindex period were included. Given the negative effect on skeletal health, glucocorticoid medications prescribed in the preindex period were included. Relevant to BrCa and skeletal health, hormonal therapies were identified from the preindex period to the day of each person's censoring date, and included selective estrogen receptor modulators (SERMs; tamoxifen and fulvestrant), aromatase inhibitors (anastrozole, letrozole, exemestane), and ovarian function suppressers (goserelin acetate, leuprolide acetate, triptorelin pamoate). Raloxifene is a type of SERM often prescribed to women with BrCa for osteoporosis management, and was therefore placed in the osteoporosis group and not in the SERM group in the current study. Baseline comorbidities were identified in the 12‐month preindex period by at least one claim with an ICD‐9‐CM code for osteoporosis, type 2 diabetes, and renal disease (i.e., acute kidney failure, renal sclerosis, or chronic kidney disease stages 1–5, dialysis, and/or kidney transplant), as described.^(^ ^28^, ^29^, ^30^, ^31^ ^)^

### Propensity‐score matching

One‐year incidence of NTFx is a rare event and may limit the interpretability of covariate‐adjusted regression models, especially for observational study designs. Therefore, to account for confounders that may influence the associations of interest without the need for adjustment of several covariates, the two comparison groups (i.e., BrCa+/CIPN−, BrCa−/CIPN−) were each matched to the primary group of interest, BrCa+/CIPN+, using a propensity score computed by the PSMATCH procedure in SAS version 9.4 (SAS Institute, Inc., Cary, NC, USA). The greedy nearest neighbor method was used after randomizing the order of the comparator groups. Given the small sample size and rare outcome, the goal of the matching ratio of 1:x (case:control) was to maximize the number of comparators per case to increase confidence in the analyses using a standard caliper of no more than 0.50, without losing any participants from the BrCa+/CIPN+ group to limit bias, while achieving success in balanced matching. For both comparison groups, age, race, US region of residence, osteoporosis, glucocorticoid medication use, and all comorbidities were initially considered to create the propensity score. For the BrCa+/CIPN− comparison group, cancer‐related variables were additionally considered to initially create the propensity score, including whether the BrCa was malignant, exposure to chemotherapy agents, and exposure to hormonal therapy (i.e., SERM, aromatase inhibitors, ovarian function suppressors). Balance in matching was examined and if not met, alternative approaches were taken (discussed below in Sensitivity analysis).

### Statistical analysis

Descriptive characteristics were summarized for each group and compared using the chi‐square test for categorical variables or the independent *t* test for continuous variables to ensure balance in matching. The crude incidence rate (IR) and 95% confidence interval (CI) of any NTFx was estimated for each group as the number of NTFx events divided by the number of person‐years and expressed as per 100 per year. The crude IR ratio (IRR) and 95% CI^(^ ^32^ ^)^ of any NTFx was estimated for the BrCa+/CIPN+ group and compared to BrCa+/CIPN−, and BrCa−/CIPN− separately. There were too few NTFx events in the primary group of interest to examine site‐specific effects.

### Sensitivity analysis

If relevant covariates were unable to achieve balance in matching or were statistically significantly different between groups, Cox proportional hazards regression was used to adjust for those covariates by estimating the hazard ratio (HR) (with 95% CI), with the interpretation focused on the main effect of group (i.e., BrCa+/CIPN+ vs. comparison group). These analyses were performed to determine if any unbalance in covariates impacted the association between group and incidence of any NTFx in the primary analysis.

Because of the observational design, results are subject to bias due to unmeasured confounding. To assess the magnitude of unmeasured confounding, the E‐value (with 95% CI) for the association between group and incidence of any NTFx was estimated. An E‐value measures the minimum strength of association needed to explain away an association between the exposure (group) and outcome (incidence of any NTFx).^(^ ^33^ ^)^

Analyses were performed using SAS version 9.4 (SAS Institute, Inc.) and *p* < 0.05 was considered statistically significant.

## Results

Balanced matching without omitting any participant from the BrCa+/CIPN+ group was not possible for BrCa+/CIPN− when malignant BrCa was included or when SERM, aromatase inhibitors, and ovarian function suppressors were included as separate variables. After omitting malignant BrCa and creating a single binary variable (yes vs. no) for SERM, aromatase inhibitors, and/or ovarian function suppressors, successful matching was achieved. For both comparison groups, the matching ratio was 1:11 (case:control). Descriptive characteristics of women with BrCa+/CIPN+ (*n* = 359) and their matched comparison groups, BrCa+/CIPN− (*n* = 3949), and BrCa−/CIPN− (*n* = 3949) is presented in Table 1. Using the chi‐square test, the prevalence of malignant BrCa was significantly lower (*p* < 0.001) and the prevalence of aromatase inhibitors was significantly higher (*p* = 0.033) for BrCa+/CIPN+ as compared to BrCa+/CIPN−; no other group differences were observed.

**Table 1:** Descriptive characteristics of propensity score matched women with (+) or without (−) BrCa and CIPN

Characteristic	BrCa+/CIPN+ (*n* = 359)	BrCa+/CIPN− (*n* = 3949)	BrCa−/CIPN− (*n* = 3949)
Age (years), mean ± SD	61.3 ± 12.1	61.3 ± 12.9	61.7 ± 12.0
18–40 years, *n* (%)	15 (4.2)	193 (4.9)	152 (3.9)
41–64 years, *n* (%)	204 (56.8)	2167 (54.9)	2217 (56.1)
≥65 years, *n* (%)	140 (39.0)	1589 (40.2)	1580 (40.0)
Race, *n* (%)			
White	251 (69.9)	2828 (71.6)	2807 (71.1)
Black	27 (7.5)	254 (6.4)	287 (7.3)
Hispanic	24 (6.7)	292 (7.4)	282 (7.1)
Asian	12 (3.3)	116 (2.9)	101 (2.6)
Other/unknown	45 (12.5)	459 (11.6)	472 (12.0)
US region of residence, *n* (%)			
West	117 (32.6)	1280 (32.4)	1200 (30.4)
Midwest	88 (24.5)	854 (21.6)	972 (24.6)
South	125 (34.8)	1511 (38.3)	1271 (32.2)
Northeast	29 (8.1)	304 (7.7)	506 (12.8)
Malignant BrCa	258 (71.9)^a^	3814 (96.6)	0 (0)
Chemotherapy, *n* (%)			
Any chemotherapy^b^	155 (43.2)	1692 (42.9)	0 (0)
Neurotoxic chemotherapy agents	139 (38.7)	1442 (36.5)	0 (0)
Comorbidities, *n* (%)			
Osteoporosis	31 (8.6)	278 (7.0)	278 (7.0)
Type 2 diabetes	67 (18.7)	681 (17.2)	714 (18.1)
Renal disease	21 (5.9)	195 (4.9)	201 (5.1)
Medication and hormonal therapy, *n* (%)			
Osteoporosis medications^c^	28 (7.8)	289 (7.3)	261 (6.6)
Glucocorticoid medications	65 (18.1)	772 (19.6)	743 (18.8)
Selective estrogen receptor modulators^c^	49 (13.7)	654 (16.6)	0 (0)
Aromatase inhibitors	122 (34.0)^a^	1131 (28.6)	0 (0)
Ovarian function suppression	^d^	26 (0.7)	0 (0)

Abbreviations: BrCa, breast cancer; CIPN, chemotherapy‐induced peripheral neuropathy.

^a^ *p* < 0.05 compared to BrCa+/CIPN−.

^b^ Any chemotherapy includes non‐neurotoxic and neurotoxic chemotherapy agents.

^c^ Raloxifene is included in the osteoporosis medication group and not the selective estrogen receptor modulators group.

^d^ *n* ≤ 10 events and not reported to maintain patient deidentification and group comparisons not performed.

The mean (standard deviation) follow‐up time in days was similar among the three groups and was 356 to 362 (29–39) days. Only one participant (BrCa+/CIPN+) from the full sample was right censored due to a drop in health plan enrollment. The crude IR and IRR of any NTFx is presented in Table 2. The crude IR (95% CI) was 4.54 (2.32–6.77) for BrCa+/CIPN+, 2.53 (2.03–3.04) for BrCa+/CIPN−, and 1.76 (1.35–2.18) for BrCa−/CIPN−. The crude IRR was significantly elevated for BrCa+/CIPN+ as compared to BrCa+/CIPN− (IRR = 1.80; 95% CI, 1.06–3.05) and BrCa−/CIPN− (IRR = 2.58; 95% CI, 1.50–4.44).

**Table 2:** IR and IRR of NTFx for women with (+) or without (−) BrCa and CIPN

Parameter	NTFx cases (*N*)	Person years	Crude IR *N* per 100/years (95% CI)	Crude IRR IRR (95% CI)	Crude IRR IRR (95% CI)
BrCa+/CIPN+	16	352	4.54 (2.32–6.77)	1.80 (1.06–3.05)	2.58 (1.50–4.44)
BrCa+/CIPN−	99	3903	2.53 (2.03–3.04)	Reference	–
BrCa−/CIPN−	69	3912	1.76 (1.35–2.18)	–	Reference

Abbreviations: BrCa, breast cancer; CI, confidence interval; CIPN, chemotherapy‐induced peripheral neuropathy; IR, incidence rate; IRR, incidence rate ratio; NTFx, nontrauma fracture.

### Sensitivity analysis

The HR of NTFx incidence before and after adjusting for covariates that were significantly different between BrCa+/CIPN+ and BrCa+/CIPN− is presented in Table 3. There were too few NTFx events to adjust for malignant BrCa. The HR remained unchanged after adjusting for aromatase inhibitors (HR = 1.79; 95% CI, 1.06–3.04).

**Table 3:** Adjusted HR of NTFx for women with (+) or without (−) BrCa and CIPN

Parameter	Unadjusted HR (95% CI)	Model 1 HR (95% CI)
BrCa+/CIPN+	1.79 (1.06–3.04)	1.79 (1.06–3.04)
BrCa+/CIPN−	Reference	Reference

*Note*: Model 1 adjusted for aromatase inhibitors.

Abbreviations: BrCa, breast cancer; CI, confidence interval; CIPN, chemotherapy‐induced peripheral neuropathy; HR, hazard ratio; NTFx, nontrauma fracture.

The E‐value (lower 95% CI) that would be needed to fully explain away the effect of BrCa+/CIPN+ compared to BrCa+/CIPN− and BrCa−/CIPN− was 3.00 (1.31) and 4.60 (2.37), respectively.

## Discussion

The main finding from this study suggests that CIPN is an independent risk factor for 1‐year NTFx incidence rate in BrCa survivors. More specifically, women with BrCa and CIPN exhibit an increased 1‐year incidence rate of NTFx compared to women with BrCa but without CIPN and women with neither. Importantly, BrCa survivors with CIPN continued to demonstrate a higher 1‐year NTFx incidence rate compared to BrCa survivors without CIPN after adjustment for aromatase inhibitor use. Collectively, our study provides evidence that CIPN may be an unrecognized risk factor for NTFx in BrCa survivors, a finding which may help to inform clinicians and guidelines aimed at optimizing bone health and fall prevention in BrCa survivors.

Prior research investigating the association between CIPN and NTFx risk in BrCa survivors is rare. However, chemotherapy has been associated with decreased bone density with the greatest evidence coming from premenopausal survivors,^(^ ^34^ ^)^ where the chemotherapy treatment induces menopause and, thereby, increases bone resorption through diminished estrogen signaling.^(^ ^35^ ^)^ In the current study, we have accounted for chemotherapy exposure between BrCa+/CIPN+ and BrCa+/CIPN− with our propensity matching study design and the median age of survivors in our study is 61 years, which is well over the median age of menopause at 51 years.^(^ ^36^ ^)^

The mechanism(s) by which CIPN may contribute to NTFx risk are unknown. However, potential mechanisms may stem from direct and indirect effects on bone mass and architecture in addition to increasing the rate of falls. Bone metabolism and specifically bone resorption can be influenced by pro‐inflammatory cytokines, which are known to be increased in CIPN,^(^ ^37^ ^)^ and may cause decrements in bone quality.^(^ ^38^ ^)^ It is proposed that extracellular matrix (ECM) protein dephosphorylation occurs with bone resorption and may contribute to fragility fractures.^(^ ^39^ ^)^ Osteopontin (OPN) is a noncollagenous protein that is abundant in the ECM of bone and whose degree of phosphorylation can influence both bone mineralization^(^ ^40^ ^)^ and the mechanical dissipation of bone impact force.^(^ ^39^, ^41^ ^)^ Interestingly, serum OPN levels have been found to be associated with the development of CIPN^(^ ^42^ ^)^ and separately with increased osteoporotic changes in postmenopausal women.^(^ ^43^ ^)^ Further research is needed to determine if ECM bone proteins, including OPN, play a role in NTFx risk among individuals with CIPN.

CIPN may also negatively influence bone mass and architecture indirectly through reductions in physical activity. Physical activity, including walking, may be decreased due to neuropathic pain or fear of falling. Adequate physical activity is known to be necessary for maintaining bone mineral density (BMD)^(^ ^44^ ^)^ and preventing sarcopenia,^(^ ^45^ ^)^ but also because it has been independently associated with a lower risk for total fractures.^(^ ^46^ ^)^ Furthermore, alterations in the response to perturbation (e.g., slips and trips) in the setting of suboptimal neuromuscular function from CIPN may cause abnormal bone loading and lead to microscopic cracks in the cortical bony architecture,^(^ ^47^ ^)^ thereby increasing the susceptibility to an eventual NTFx.^(^ ^48^, ^49^ ^)^

Falls biomechanically challenge the fracture resistance capability of bone and may increase risk for NTFx.^(^ ^50^ ^)^ One model of falls^(^ ^51^ ^)^ suggests that falls result due to an inability to successfully respond to perturbation, which is a function of both neuromuscular^(^ ^52^ ^)^ and neurocognitive^(^ ^53^ ^)^ capacities. Previous research has demonstrated that CIPN's neuromuscular dysfunction, including changes in sensation, postural instability,^(^ ^54^ ^)^ and strength,^(^ ^55^ ^)^ cause gait impairments in BrCa survivors^(^ ^11^ ^)^ and lead to falls.^(^ ^9^, ^10^ ^)^ Recently, depression^(^ ^56^ ^)^ and anxiety,^(^ ^57^ ^)^ known fall risk factors, have been associated with BrCa survivors with CIPN.^(^ ^58^ ^)^ Moreover, alterations in neurocognitive function impact gait impairments^(^ ^59^ ^)^ among BrCa survivors with CIPN and likely contribute to increased fall risk in this vulnerable population.

Our study is not without limitations and many are secondary to the use of claims data, which is dependent on patients reporting their symptoms and providers selecting the proper codes for diagnoses, medications, and procedures. Therefore, the prevalence of CIPN, chemotherapeutic use, and other medication use are likely underrepresented approximations. Due to a likely underrepresentation of chemotherapeutic use and use of an ICD‐9 code, we are unable to determine if the peripheral neuropathy is truly due to chemotherapy. However, it has been shown that claims data shows high specificity for CIPN.^(^ ^60^ ^)^ Due to lack of imaging correlation of the fractures and survivor's history of cancer we are unable to completely attribute the fractures to insufficiency and not to metastasis or trauma. Our study is also limited as claims data do not contain information on physical activity, BMD, Fracture Risk Assessment Tool (FRAX) score, or BMI, which are all known variables impacting NTFx risk in breast cancer survivors. Last, this study included fractures without evidence of trauma. Previous studies have found that individuals with low BMD also sustain a higher rate of high‐trauma fractures.^(^ ^61^, ^62^ ^)^ Future studies are needed to determine if CIPN is associated with high‐trauma fractures.

These limitations notwithstanding, our findings may have clinical implications. Specifically, we have identified CIPN as a novel risk factor in BrCa survivors for NTFx, independent of aromatase inhibitor use. Currently, guidelines use BMD, evaluated by dual‐energy x‐ray absorptiometry (DXA) scan, to clinically evaluate risk of NTFx and prescription of bone modifying agents. Despite added benefit, the use of BMD does not always identify at risk populations, including BrCa survivors who suffer fractures at a higher BMD than women with postmenopausal osteoporosis.^(^ ^63^ ^)^ Given the morbidity and mortality associated with NTFx, it is essential to identify BrCa survivors at risk for NTFx. If other studies confirm CIPN to be a risk factor for NTFx, then its addition to the ASCO and ESMO guidelines for bone health in BrCa survivors may be warranted.

In summary, our study provides evidence that CIPN is an independent risk factor for NTFx in BrCa survivors. Moving forward, research should focus on validating the association of CIPN with NTFx because it may inform future clinical decision making and future guidelines aimed at managing bone fragility in BrCa survivors. Future research is necessary to elucidate the precise mechanism(s) responsible for the association we found between CIPN and NTFx. Overall, BrCa survivors with CIPN may benefit from rehabilitative interventions that mitigate their fall risk and optimize their bone health.

## Disclosures

Daniel G. Whitney receives funding through the University of Michigan Office of Health Equity and Inclusion Diversity Fund and the American Academy for Cerebral Palsy and Developmental Medicine. James K. Richardson received funding from the Newman Family Foundation for the open access publication of this manuscript. The authors Sarah G. Bell and Brendan L. McNeish did not receive support from any organization. The authors (Brendan L. McNeish, James K. Richardson, Sarah G. Bell, and Daniel G. Whitney) do not have any conflicts of interests for the submitted work.

## Author contributions

Brendan L. McNeish Conceptualization; data curation; formal analysis; investigation; methodology; project administration; validation; writing‐original draft; writing‐review & editing. James K. Richardson: Conceptualization; formal analysis; investigation; resources; supervision; writing‐review & editing. Sarah G. Bell: Conceptualization; investigation; methodology; writing‐review & editing. Daniel G. Whitney: Conceptualization; data curation; formal analysis; investigation; methodology; project administration; software; supervision; validation; writing‐review & editing.

## Availability of data

The data that support the findings of this study are available from Optum's De‐identified Clinformatics® Data Mart Database (OptumInsight, Eden Prairie, MN, USA), but restrictions apply to the availability of these data, which were used under license for the current study, and so are not publicly available. Data are, however, available from the authors upon reasonable request and with permission of Optum's De‐identified Clinformatics Data Mart Database (OptumInsight).

## Availability of code

Data are available from the authors upon reasonable request.

### Peer review

The peer review history for this article is available at https://publons.com/publon/10.1002/jbm4.10519.

## Supporting information


**Supplementary Table S1** Diagnostic and chemotherapy codes used.
