# Supplementary material for: Chemotherapy‐induced peripheral neuropathy increases nontraumatic fracture risk in breast cancer survivors
Source: JBMR Plus. 2021 Jun 10;5(8):e10519. doi: 10.1002/jbm4.10519 (PMC8328798; doi:10.1002/jbm4.10519)
Supplement: Supplementary file 1 — Supplementary Table S1 Diagnostic and chemotherapy codes used. [file JBM4-5-e10519-s001.doc]

**Supplementary Table 1**- Diagnostic and Chemotherapy Codes Used

| Code Type | Code | General Classification | Description |
| --- | --- | --- | --- |
| ICD-9 | 174.1-174.9 | Breast Cancer | Malignant Breast Cancer |
| ICD-9 | 233.0 | Breast Cancer | Non-malignant Breast cancer |
| ICD-9 | 357.6 | Polyneuropathy | Polyneuropathy due to Drugs |
| HCPCS | J9171 | Neurotoxic Chemotherapy | Docetaxel |
| HCPCS | J9264, J9265, J9267 | Neurotoxic Chemotherapy | Paclitaxel |
| HCPCS | J9045 | Neurotoxic Chemotherapy | Carboplatin |
| HCPCS | J9060 | Neurotoxic Chemotherapy | Cisplatin |
| HCPCS | J9263 | Neurotoxic Chemotherapy | Oxaliplatin |
| HCPCS | J9360 | Neurotoxic Chemotherapy | Vinblastine |
| HCPCS | J9370, J9371 | Neurotoxic Chemotherapy | Vincristine |
| HCPCS | J9390 | Neurotoxic Chemotherapy | Vinorelbine |
| HCPCS | C9240, J9207 | Neurotoxic Chemotherapy | Ixabepilone |
| HCPCS | J9179, C9280 | Neurotoxic Chemotherapy | Eribulin mesylate |
| HCPCS | J9041 | Neurotoxic Chemotherapy | Bortezomib |
| HCPCS | J9000-2, Q2048-50 | Chemotherapy | Doxorubicin |
| HCPCS | J8610, J9250, J9260 | Chemotherapy | Methotrexate |
| HCPCS | J8520-1 | Chemotherapy | Capecitabine |
| HCPCS | J9190 | Chemotherapy | Fluorouracil |
| HCPCS | J9091-2, J9094, J9097, J9070, J8530 | Chemotherapy | Cyclophosphamide |
| HCPCS | J9181, J8560 | Chemotherapy | Etoposide |
| HCPCS | J9201 | Chemotherapy | Gemcitabine |
